# Differences of left ventricular systolic deformation in hypertensive patients with and without apical hypertrophic cardiomyopathy

**DOI:** 10.1186/1476-7120-11-40

**Published:** 2013-11-14

**Authors:** Yu-Cheng Kao, Ming-Feng Lee, Chun-Tai Mao, Wei-Siang Chen, Ning-I Yang, Wen-Jin Cherng, Ming-Jui Hung

**Affiliations:** 1 From the Cardiology Section, Department of Medicine, Chang Gung Memorial Hospital, Keelung, 222 Maijin Road, Keelung 20401, Taiwan; 2 Chang Gung University College of Medicine, Taoyuan, Taiwan

**Keywords:** Hypertrophic cardiomyopathy, Echocardiography, Left ventricular function

## Abstract

**Background:**

We tested the hypothesis that the apical myocardial mechanics differ from those of other ventricular segments in hypertensive patients with and without apical hypertrophic cardiomyopathy (ApHCM).

**Methods:**

We retrospectively studied hypertensive patients with and without ApHCM. Left ventricular longitudinal, circumferential, and radial strains were examined by two-dimensional speckle-tracking echocardiography at the basal, middle, and apical walls of the parasternal short-axis and apical 2-, 3- and 4-chamber views.

**Results:**

Fourteen consecutive patients with hypertension and ApHCM and 14 patients with hypertension without ApHCM were studied. Lower mitral annular peak systolic velocity and greater diastolic dysfunction were present in hypertensive patients with ApHCM than in hypertensive patients without ApHCM. Compared with hypertensive patients without ApHCM, hypertensive patients with ApHCM had significantly lower apical longitudinal (−13.9% vs −21.9%, p = 0.010) and radial strains (4.4% vs 11.5%, p = 0.017) without the base-to-apex gradient. The global longitudinal (−15.6% vs −18.8%, p = 0.027) and circumferential strains (−16.1% vs −19.2%, p = 0.019) were significantly lower in hypertensive patients with ApHCM than in hypertensive patients without ApHCM. Among systolic parameters, the global longitudinal strain was independently associated with hypertension with ApHCM (odds ratio, 1.457; 95% confidence interval, 1.002–2.119; p = 0.049).

**Conclusions:**

Reduced apical longitudinal and radial strains without a base-to-apex gradient were present in hypertensive patients with ApHCM. The global longitudinal strain was independently associated with ApHCM in hypertensive patients.

## Introduction

Apical hypertrophic cardiomyopathy (ApHCM), in which the myocardial wall thickening is localized at the apex of the left ventricle, is comparatively rare in western countries in comparison with Asia [1,2]. ApHCM is described as an electrocardiographic pattern of giant negative T waves and an angiographic feature of end-diastolic left ventricular cavity structure resembling an ‘ace of spades’ [3,4]. It has a benign clinical course in terms of cardiovascular mortality [1,2,5,6]; however, it may be associated with serious complications, such as myocardial infarction and arrhythmias [2,6]. Less benign clinical outcomes of patients with ApHCM and hypertension have been observed recently [7]. The presentation of ApHCM in the setting of chronic hypertension is recognized as an important issue because an accurate diagnosis has an impact on prognosis and management [8].

Echocardiographic strain imaging is an innovative approach recently developed for the assessment of left ventricular myocardial mechanics [9]. Myocardial strain can be determined using tissue Doppler imaging or two-dimensional speckle tracking. Doppler-based techniques are limited by the angle-dependence of the signal, precluding the assessment of apical left ventricular function. In contrast, two-dimensional speckle tracking studies orthogonal components of strain independent of the insonation angle because it tracks deformation between acoustic markers in the ultrasonic image in two dimensions [9]. Few studies have addressed regional myocardial mechanics in patients with ApHCM [10-12], and 2-dimensional deformation imaging studies of hypertensive patients with and without ApHCM are lacking. For the present study, we hypothesized that regional myocardial mechanics of the apex differed from those of the other ventricular segments in hypertensive patients with and without ApHCM. Therefore, in this retrospective 2-dimensional echocardiographic study, we investigated left ventricular deformation in hypertensive patients with and without ApHCM.

## Methods

### Study population

The study patients were identified through the cardiac care outpatient clinic of Chang Gung Memorial Hospital, Keeling, a tertiary referral hospital. We retrospectively studied the clinical outcomes of consecutive patients with a new diagnosis of hypertension and ApHCM between August 2011 and September 2012. Data were abstracted on demographic characteristics, coronary risk factors, symptoms, and findings on physical examination at the time of presentation, and diagnosis of ApHCM, as well as the most recent follow-up visit. The control group consisted of age-matched asymptomatic patients who had hypertension, but no ApHCM. The study was approved by the Research Ethics Review Board of Chang Gung Memorial Hospital (101-2038B).

### Diagnostic criteria

The echocardiographic inclusion criteria for ApHCM were the following: 1) asymmetric left ventricular hypertrophy confined primarily to the left ventricular apex below the papillary muscle level; 2) apical wall thickness ≥ 15 mm; 3) a ratio of maximal apical to posterior wall thickness ≥ 1.5. Patients were excluded if they had one of following: 1) a severe valvular lesion; 2) sustained atrial or ventricular arrhythmias; 3) prior percutaneous intervention; 4) prior cardiac surgery; 5) prior myocardial infarction; 6) pericardial disease; 7) immunological disease; 8) active infection; 9) moderate to severe anemia or 10) hyper- or hypothyroidism.

### Clinical data

Current smoking status was defined as having smoked at least half of a pack of cigarettes per year and having smoked at least one cigarette within 3 weeks before enrollment. Diabetes mellitus was defined as a fasting glucose level ≥ 126 mg/dL or use of hypoglycemic medication. Hypercholesterolemia was defined as a low-density lipoprotein level ≥ 130 mg/dL in a fasting blood sample or use of a statin medication. Hypertension was defined as use of antihypertensive medications or a blood pressure > 140/90 mmHg. Ischemic heart disease was confirmed by 1) coronary angiography, with ≥ 50% diameter stenosis in one or more coronary vessels after administration of intracoronary nitroglycerin or 2) a ^201^thallium myocardial perfusion scan showing reversible/irreversible perfusion defects. Glomerular filtration rate was estimated using the Modification of Diet in Renal Disease Study 4-variable equation [13].

### Electrocardiography

Electrocardiograms were analyzed for the presence of left ventricular hypertrophy according to the Sokolow-Lyon criteria [14]. The corrected QT interval was measured in lead V_2_[15]. ‘Giant’ T wave negativity was defined as a negative T wave voltage ≥ 1 mV (≥ 10 mm) in any of the leads.

### Standard echocardiography

All echocardiograms were performed by two experienced physicians (M-J and N-I) who used a commercially available system (Vivid E9, General Electric-Vingmed, Milwaukee, Wisconsin). Images were obtained with patients in the left lateral decubitus position at end-expiration. All standard measurements were obtained in the parasternal long- and short-axis views; apical 4-chamber, 2-chamber, and long-axis views. Two-dimensional and color Doppler imaging were performed to screen for wall motion abnormalities, mitral annulus calcification, and valvular stenosis or regurgitation. For pulsed-tissue Doppler studies, a 2-mm sampling volume was used from the apical 4-chamber view in the septal mitral annulus. The maximal apical wall thickness was obtained as the average of the measurements in the apical 4-chamber and 2-chamber views at end-diastole. Left ventricular ejection fraction and stroke volume were obtained by quantitative 2-dimensional ultrasonography as previously described [16]. Transmitral pulsed-wave Doppler and tissue Doppler were recorded in the apical 4-chamber view. Pulsed-wave Doppler velocities of the pulmonary venous flow were obtained in the right upper pulmonary vein. Tissue Doppler imaging of the septal mitral annulus was used to measure mitral annular velocities in peak systole (Sm) and in early (Em) and late diastole (Am). Diastolic function was categorized as: normal, impaired relaxation, pseudonormalized filling, and restrictive filling [17]. The time interval from the end to the onset of the mitral annular velocity pattern during diastole (a_m_) and the duration of the S-wave (b_m_) were measured and used to calculate the myocardial performance index as (a_m_-b_m_)/b_m_[18]. The isovolumic relaxation time (IVRT) was calculated as the time interval between Sm and Em, and the isovolumic contraction time was calculated as the time interval between Am and Sm.

### Two-dimensional speckle-tracking echocardiography

Two-dimensional strain analysis was performed offline using Echopac software, version 110.1.2 (General Electric-Vingmed) by two independent observers who were unaware of the patients’ conditions. All strain images were obtained at a frame rate of 60–90 frames/s. For each of the three short-axis views, the sampling points were placed manually along the endocardium at the left ventricular base, middle, and apex during end-systole. For each of the 2-, 3- and 4-chamber views, three sampling points were placed manually at the septal mitral annulus, lateral corner, and apical endocardium. A region of interest was then generated by the software covering the myocardial thickness along the entire left ventricular wall. The region of interest was adjusted manually to ensure that the inner margin conformed to the entire left ventricular endocardial border and that it included the entire thickness of the left ventricular myocardium. The software subsequently identified the tissue speckles and tracked their movement frame-by-frame throughout the cardiac cycle.

The left ventricular wall was divided into six segments arranged circumferentially at the basal, middle, and apical levels. The software algorithm then calculated longitudinal, circumferential, and radial strains for each segment in graphical form, with automated measurements recorded in tabular form. Peak systolic longitudinal, circumferential, and radial strains for each segment were recorded: the values for all the myocardial segments for each patient were averaged to obtain the global values. End-systole was defined by the time of aortic valve closure and end-diastole was defined by the time of mitral valve closure by Doppler ultrasonography from the apical 4-chamber view.

### Reproducibility

Inter- and intra-observer variability were assessed by two experienced physicians evaluating the raw data of five patients with hypertension and five patients with hypertension and ApHCM in a blinded manner at baseline and at 1 week later. They were instructed to measure strain parameters independently from each other.

### Statistical analyses

The sample size calculation was based on the differences in the mean between the two groups with equal sample size, pre-specified 5% type I error, and 90% power (Z_1-β_ = 1.00). We performed a sample size calculation using Power Analysis Statistical Software (PASS 6.0, license 13451701; NCSS Inc., Kaysville, Utah). A sample size of 12 subjects in the control and ApHCM groups would achieve 90% power to detect a difference of 20% strain between the null hypothesis that both group differences in the means are 0.00 and the alternative hypothesis that the differences in means between the two groups is 20% strain, with a 2-sided test and a significance level of 0.05.

Continuous variables with skewed distributions and *p* values of < 0.05 by Kolmogorov-Smirnov testing were presented as medians (25th, 75th percentiles), and those not skewed were expressed as means ± standard deviations. For normally distributed continuous variables, a two-sample unpaired *t*-test was performed. For variables with skewed distributions, the Wilcoxon rank sum test and Fisher’s exact test were used. Receiver-operating characteristic curves were constructed, and areas under curve were calculated. Sensitivities and specificities were determined for the ability to identify ApHCM. Reproducibilities (both interobserver and intra-observer variability) of speckle-tracked echocardiographic parameters were tested using the Bland-Altman statistic. A *p*-value of < 0.05 was considered statistically significant. Statistical analyses were performed using SPSS software version 15.0 for Windows (Chicago, Illinois).

## Results

### Clinical and electrocardiographic characteristics

**Figure 1:**
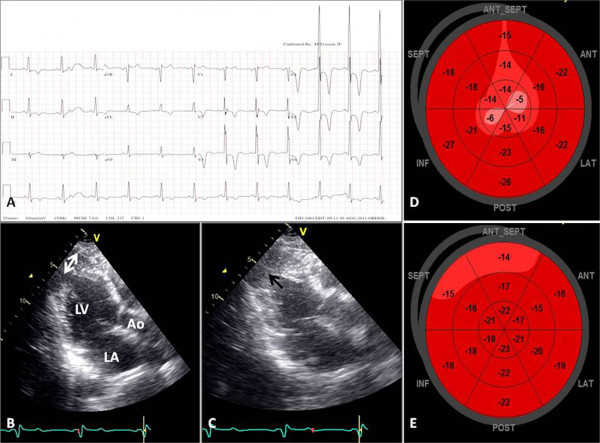
**A representative patient with hypertension and ApHCM. (A)** The 12-lead resting electrocardiogram shows left ventricular hypertrophy with a giant negative T-wave in the V_2–4_ leads. Parasternal long-axis echocardiographic images show apical hypertrophy (double arrowheads) at the end-diastolic phase **(B)** and apical obstruction (arrow) at the end-systolic phase **(C)**. Representative examples of global longitudinal strain (GLS) images in hypertensive patients with ApHCM **(D, GLS = −17.3%)** and without ApHCM **(E, GLS = −18.3%)**. The GLS decreases without a base-to-apex gradient in hypertensive patients with ApHCM as compared with hypertensive patients without ApHCM.

Fourteen consecutive patients with hypertension and ApHCM and 14 patients with hypertension without ApHCM were studied. The clinical and electrocardiographic characteristics of the two groups are compared in Table 1. Nearly half of the hypertensive patients with ApHCM presented with dyspnea on exertion and one-fifth of the patients were asymptomatic. All of the hypertensive patients with ApHCM experienced mild-to-moderate dyspnea (New York Heart Association functional class < 3). As compared with hypertensive patients without ApHCM, hypertensive patients with ApHCM had a higher body mass index, lower diastolic blood pressure, and lower estimated glomerular filtration rate. Hypertensive patients with ApHCM had higher prevalences of left ventricular hypertrophy and giant negative T wave (Figure 1A), and larger maximal T wave inversion than in the hypertensive patients without ApHCM.

**Table 1:** Comparison of clinical and electrocardiographic characteristics between hypertensive patients with and without apical hypertrophic cardiomyopathy (ApHCM)

	**Hypertension with ApHCM (n = 14)**	**Hypertension without ApHCM (n = 14)**	**p**
Age (years)	62 ± 11	63 ± 12	0.784
Female (%)	36	57	0.449
BMI (kg/m^2^)	30 (27, 32)	25 (23, 28)	0.013
Systolic blood pressure, mmHg	130 ± 12	138 ± 10	0.069
Diastolic blood pressure, mmHg	70 ± 8	77 ± 7	0.030
Heart rate (beats/min)	66 ± 11	67 ± 10	0.740
Symptoms at presentation (%)			
Chest pain	29		
Palpitation	7		
Syncope	0		
Dyspnea on exertion	43		
Asymptomatic	21		
NYHA class at presentation (%)			
I	43		
II	57		
III and IV	0		
Smoking (%)	50	43	1.000
Diabetes (%)	29	21	1.000
Ischemic heart disease (%)	21	14	1.000
Dyslipidemia (%)	57	36	0.449
Estimated GFR (ml/min/m^2^)	70 ± 25	93 ± 27	0.029
Low-density lipoprotein (mg/dL)	111 ± 42	108 ± 29	0.796
Medications (%)			
β-blockers	43	43	1.000
ACEI/ARB	64	60	1.000
Calcium antagonists	86	50	0.103
Diuretics	29	7	0.326
Nitrates	21	7	0.596
Aspirin	21	36	0.678
Statins	36	36	1.000
Electrocardiography			
LV hypertrophy (%)	86	21	0.002
Giant negative T waves (%)	50	0	0.006
Maximal T wave inversion (mm)	10.3 (7.3, 15.0)	0.5 (0, 2.1)	< 0.001
Corrected QT interval (ms)	446 ± 33	428 ± 17	0.081

Data are presented as means ± SD, number, or medians (25th, 75th percentiles).

ACEI = angiotensin-converting enzyme inhibitor; ARB = angiotensin II receptor blocker; BMI = body mass index; GFR = glomerular filtration rate; LV = left ventricular hypertrophy.

### Standard echocardiography

The standard echocardiographic parameters of the two groups are compared in Table 2. Left ventricular wall thickness, apex thickness (Figure 1B, C), and left atrial diameter were greater in hypertensive patients with ApHCM than in hypertensive patients without ApHCM. Hypertensive patients with ApHCM had lower Sm, a higher myocardial performance index, longer IVRT, and greater prevalence of left ventricular diastolic dysfunction. Although lower diastolic blood pressure and larger body mass index were present in hypertensive patients with ApHCM, left ventricular systolic (lower Sm) and diastolic (dilated left atrium, longer IVRT, and higher diastolic function grade) function were more impaired in these patients than in hypertensive patients without ApHCM, which indicates that ApHCM alone had a negative impact on left ventricular systolic and diastolic function.

**Table 2:** Comparison of standard echocardiographic parameters between hypertensive patients with and without apical hypertrophic cardiomyopathy (ApHCM)

	**Hypertension with ApHCM**	**Hypertension without ApHCM**	**p**
Interventricular septum thickness (mm)	12.9 ± 2.7	10.2 ± 2.3	0.007
Posterior wall thickness (mm)	10.8 ± 2.6	8.3 ± 1.9	0.008
Apex thickness (mm)	23.8 (22.7, 29.9)	8.6 (7.8, 9.2)	< 0.001
LV end-diastolic diameter (mm)	50 ± 5	50 ± 4	0.975
LV end-systolic diameter (mm)	28 ± 8	31 ± 3	0.255
LV end-diastolic volume index (ml/m^2^)	29 ± 12	38 ± 16	0.086
LV end-systolic volume index (ml/m^2^)	9 ± 4	12 ± 7	0.201
Stroke volume index (ml/m^2^)	20 ± 9	26 ± 10	0.083
LV ejection fraction (%)	68 ± 8	69 ± 8	0.697
LA end-systolic diameter (mm)	47 ± 6	39 ± 5	0.001
Mitral E/A ratio	1.00 ± 0.50	0.82 ± 0.20	0.226
Sm (cm/s)	5.6 ± 1.5	6.9 ± 1.5	0.030
Myocardial performance index	0.76 ± 0.31	0.56 ± 0.12	0.039
IVRT (ms)	128 ± 32	100 ± 21	0.012
IVCT (ms)	68 ± 22	65 ± 18	0.681
E/Em	15.1 ± 4.3	13.0 ± 2.6	0.128
LV diastolic function, grade			0.045
Normal	0	2	
Impaired relaxation, I	10	12	
Pseudonormalized filling, II	4	0	
Restrictive filling, III	0	0	

Data are presented as means ± SD, number, or median (25th, 75th percentiles).

A = atrial contraction; D = diastolic; E = rapid filling; Em = mitral annular early diastolic velocity; IVRT = isovolumic relaxation time; IVCT = isovolumic contraction time; LA = left atrial; LV = left ventricular; Sm = mitral annular peak systolic velocity.

### Two-dimensional speckle-tracking echocardiography

**Figure 2:**
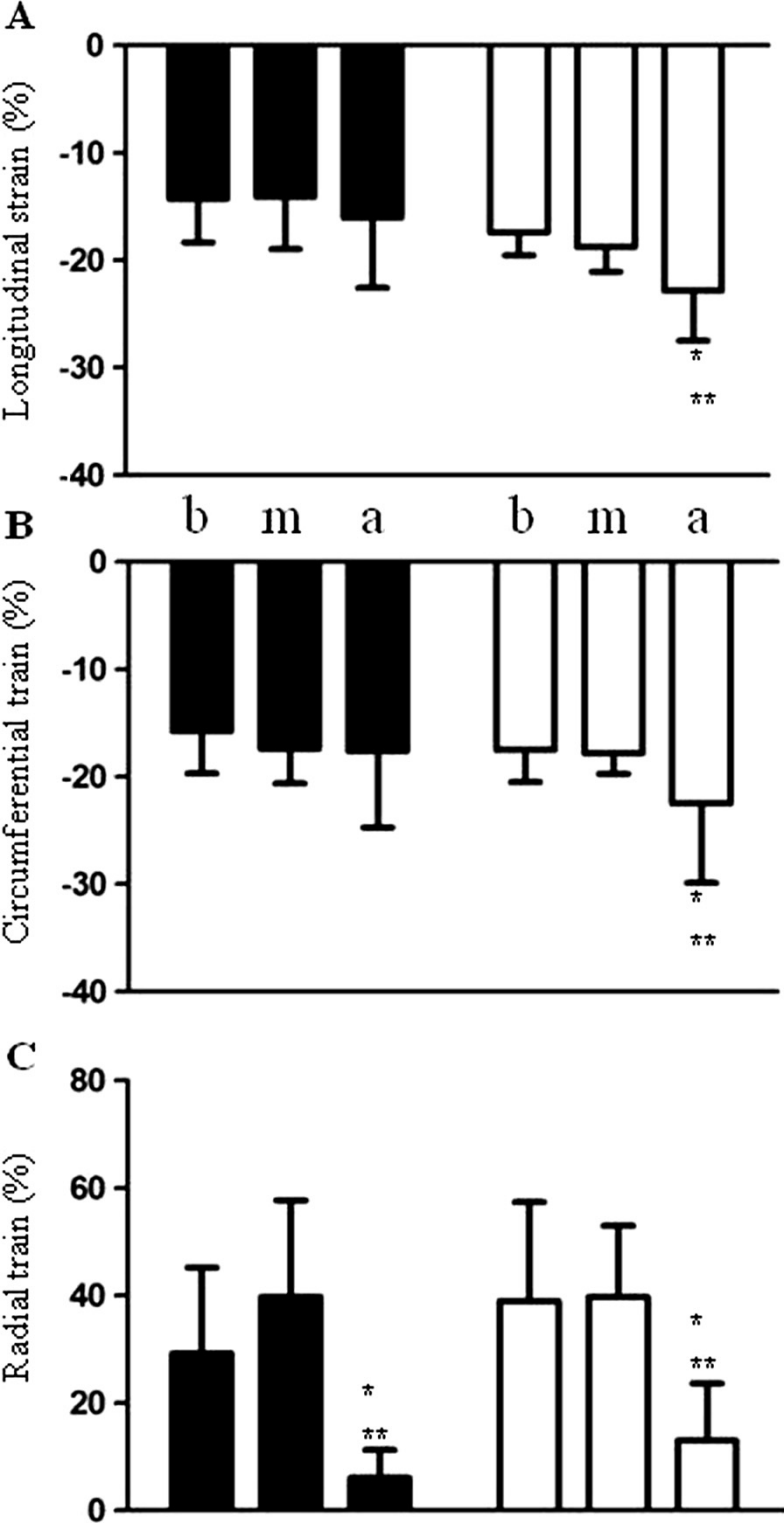
**The base-to-apex gradients of longitudinal. (A)**, circumferential **(B)**, and radial **(C)** strains are not observed in hypertensive patients with ApHCM (black bars) compared with hypertensive patients without ApHCM (white bars). a = apex; b = base; m = middle; *p < 0.05 versus b; **p < 0.05 versus m.

Table 3 depicts the speckle-tracking echocardiographic parameters of the two groups. Overall (Figure 1 D, E), the global longitudinal and circumferential strains decreased, and apical, but not basal and middle, longitudinal, and radial strains, decreased in hypertensive patients with ApHCM compared with hypertensive patients without ApHCM. From the segmental view, the apical longitudinal and radial strains consistently decreased in hypertensive patients with ApHCM compared with hypertensive patients without ApHCM. Furthermore, the apical longitudinal and radial strains in hypertensive patients with ApHCM did not show a base-to-apex gradient as in hypertensive patients without ApHCM (Figure 2). Longitudinal strain of the lateral and anterior walls decreased in all segments, whereas the longitudinal strain of the septal and inferior walls decreased only in the apical segment among hypertensive patients with ApHCM compared with hypertensive patients without ApHCM. Circumferential strain decreased only in the apical lateral and anterior walls among hypertensive patients with ApHCM compared with hypertensive patients without ApHCM. Radial strain decreased in all apical walls, except the septal and inferior walls in hypertensive patients with ApHCM compared with hypertensive patients without ApHCM. Overall, apical anterior and lateral wall strains decreased consistently irrespective of the measurement.

**Table 3:** Comparison of segmental values in each left ventricular wall for left ventricular strain in hypertensive patients with and without apical hypertrophic cardiomyopathy (ApHCM)

**Strain/segment/wall**	**Hypertension with ApHCM**	**Hypertension without ApHCM**	**p**
Longitudinal strain (%)			
Basal			
Septal	−15.8 ± 6.3	−15.8 ± 4.2	0.983
Lateral	−11.5 ± 7.0	−16.6 ± 4.7	0.029
Inferior	−18.7 ± 6.0	−20.0 ± 5.0	0.552
Anterior	−11.1 ± 4.1	−15.3 ± 4.0	0.012
Posterior	−17.1 ± 8.5	−18.9 ± 6.4	0.534
Anteroseptal	−8.7 (−6.7, -14.4)	−17.1 (−14.3, -21.7)	0.009
All walls	−14.4 (−11.8, -16.1)	−17.6 (−15.5, -19.2)	0.013
Middle			
Septal	−15.8 ± 5.8	−19.0 ± 2.9	0.071
Lateral	−11.6 ± 5.6	−16.2 ± 3.4	0.016
Inferior	−15.2 ± 10.1	−20.8 ± 3.7	0.062
Anterior	−11.8 (−6.7, -14.4)	−17.8 (−12.6, -20.8)	0.002
Posterior	−17.3 ± 7.7	−20.2 ± 4.4	0.227
Anteroseptal	−13.7 (−8.1, -17.9)	−18.7 (−13.9, -18.7)	0.016
All walls	−13.5 (−10.5, -18.8)	−18.5 (−17.4, -20.8)	0.009
Apical			
Septal	−15.0 (−12.8, -22.5)	−25.2 (−21.8, -29.2)	0.001
Lateral	−14.3 (−10.6, -20.0)	−21.1 (−17.0, -24.8)	0.005
Inferior	−12.6 (−7.2, -22.9)	−22.2 (−19.0, -27.2)	0.011
Anterior	−12.5 ± 8.5	−21.8 ± 7.6	0.005
Posterior	−18.9 ± 8.2	−22.4 ± 5.4	0.201
Anteroseptal	−17.4 ± 9.3	−22.1 ± 7.1	0.141
All walls	−13.9 (−10.6, -23.3)	−21.9 (−19.2, -27.4)	0.010
All segments	−15.6 (−12.1, -19.2)	−18.8 (−17.7, -21.9)	0.027
Circumferential strain (%)			
Basal			
Septal	−19.4 ± 9.5	−22.9 ± 6.7	0.269
Lateral	−11.0 ± 5.5	−12.9 ± 5.7	0.375
Inferior	−16.5 ± 4.5	−17.2 ± 5.5	0.685
Anterior	−14.6 ± 8.0	−16.2 ± 6.9	0.584
Posterior	−13.2 ± 5.5	−13.7 ± 6.1	0.816
Anteroseptal	−19.8 ± 9.0	−22.1 ± 4.8	0.418
All walls	−15.7 ± 3.9	−17.5 ± 3.0	0.195
Middle			
Septal	−22.5 ± 5.6	−26.2 ± 4.0	0.051
Lateral	−14.7 ± 6.2	−13.5 ± 4.8	0.560
Inferior	−18.6 ± 4.8	−17.3 ± 5.9	0.544
Anterior	−18.8 ± 6.0	−17.3 ± 6.3	0.502
Posterior	−14.5 ± 6.1	−13.4 ± 8.0	0.682
Anteroseptal	−21.9 ± 5.2	−26.3 ± 4.7	0.026
All walls	−17.4 ± 3.2	−17.8 ± 1.9	0.687
Apical			
Septal	−19.1 ± 7.2	−18.6 ± 6.9	0.863
Lateral	−16.9 ± 8.2	−25.6 ± 9.0	0.013
Inferior	−20.0 ± 8.2	−21.7 ± 6.3	0.532
Anterior	−14.5 ± 8.0	−24.7 ± 9.7	0.006
Posterior	−19.3 ± 8.5	−24.6 ± 7.4	0.093
Anteroseptal	−16.0 ± 6.4	−20.0 ± 8.1	0.160
All walls	−17.6 ± 7.1	−22.5 ± 7.4	0.085
All segments	−16.1 (−14.9, -19.5)	−19.2 (−18.0, -20.3)	0.019
Radial strain (%)			
Basal			
Septal	28.4 ± 17.1	40.2 ± 19.5	0.102
Latera	29.6 ± 17.9	39.6 ± 21.8	0.197
Inferior	29.9 ± 19.7	42.6 ± 19.9	0.101
Anterior	27.5 ± 14.3	34.3 ± 20.0	0.309
Posterior	30.8 ± 20.3	43.1 ± 21.5	0.132
Anteroseptal	29.1 ± 15.8	33.9 ± 17.6	0.457
All walls	29.2 ± 16.0	39.0 ± 18.5	0.149
Middle			
Septal	40.0 ± 17.7	40.3 ± 14.0	0.962
Lateral	40.3 ± 18.9	37.8 ± 15.5	0.706
Inferior	40.8 ± 17.5	40.0 ± 16.7	0.902
Anterior	38.3 ± 20.3	40.8 ± 16.6	0.722
Posterior	40.9 ± 18.0	38.5 ± 17.3	0.723
Anteroseptal	38.3 ± 18.5	41.1 ± 15.1	0.666
All walls	39.8 ± 18.0	39.8 ± 13.3	0.998
Apical			
Septal	6.7 ± 5.2	11.8 ± 11.7	0.147
Lateral	4.1 (1.3, 12.1)	12.8 (10.7, 17.0)	0.008
Inferior	6.4 ± 5.5	12.1 ± 11.0	0.093
Anterior	3.7 (0.8, 11.2)	12.9 (10.7, 20.9)	0.005
Posterior	4.5 (1.7, 12.4)	11.4 (9.9, 15.4)	0.024
Anteroseptal	4.0 (1.7, 10.8)	11.0 (9.2, 14.2)	0.033
All walls	4.4 (1.7, 12.2)	11.5 (10.1, 14.7)	0.017
All segments	25.0 ± 9.4	30.6 ± 7.6	0.097

Data are presented as means ± SD or median (25th, 75th percentiles).

### Association with ApHCM

**Figure 3:**
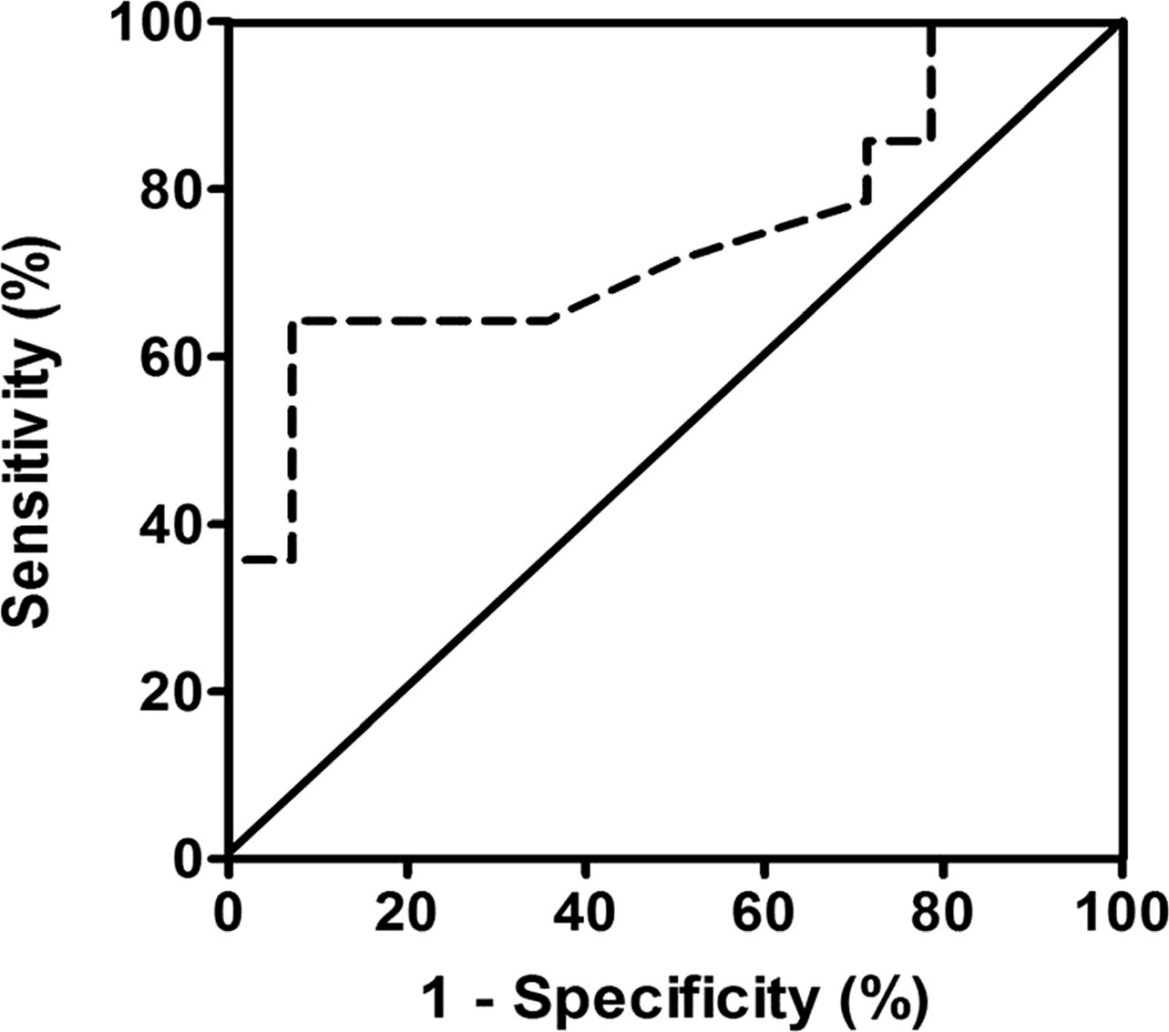
**Receiver-operator characteristic analysis.** The optimum cut-off value for left ventricular global longitudinal strain to differentiate the presence of hypertension with ApHCM was −16.8%; the sensitivity and specificity were 64% and 93%, respectively. The area under curve was 0.74 ± 0.10 (95% CI: 0.55–0.93, p < 0.029).

Our final multivariate model controlled for systolic parameters with *p* < 0.10, i.e., Sm, left ventricular global longitudinal, circumferential, and radial strains. After controlling for these covariates, multivariate analysis showed that global longitudinal strain was an independent predictor of hypertension with ApHCM (Table 4). Harrell’s C statistic of the multivariate model showed a strong discriminative power [0.862, 95% confidence interval (CI): 0.716–1.008, *p* = 0.001]. The cut-off value for left ventricular global longitudinal strain that predicted the presence of hypertension with ApHCM was > −16.8% according to receiver-operator characteristic analysis (area under curve = 0.74 ± 0.10, 95% CI: 0.55–0.93, *p* = 0.029, Figure 3). The sensitivity, specificity, positive and negative predictive values, and positive likelihood ratio for this ratio in detecting hypertension with ApHCM in our cohort were 64%, 93%, 90%, 72%, and 9.0, respectively.

**Table 4:** Multivariable logistic regression analysis of left ventricular systolic parameters for prediction of apical hypertrophic cardiomyopathy in hypertensive patients

	**Multivariate**		
	**Odds ratio**	**95% CI**	**p**
Sm	0.391	0.145-1.056	0.064
Global longitudinal strain	1.457	1.002-2.119	0.049
Global circumferential strain	1.560	0.912-2.669	0.105
Global radial strain	0.952	0.818-1.107	0.533

Sm = mitral annular peak systolic velocity.

### Reproducibility

**Figure 4:**
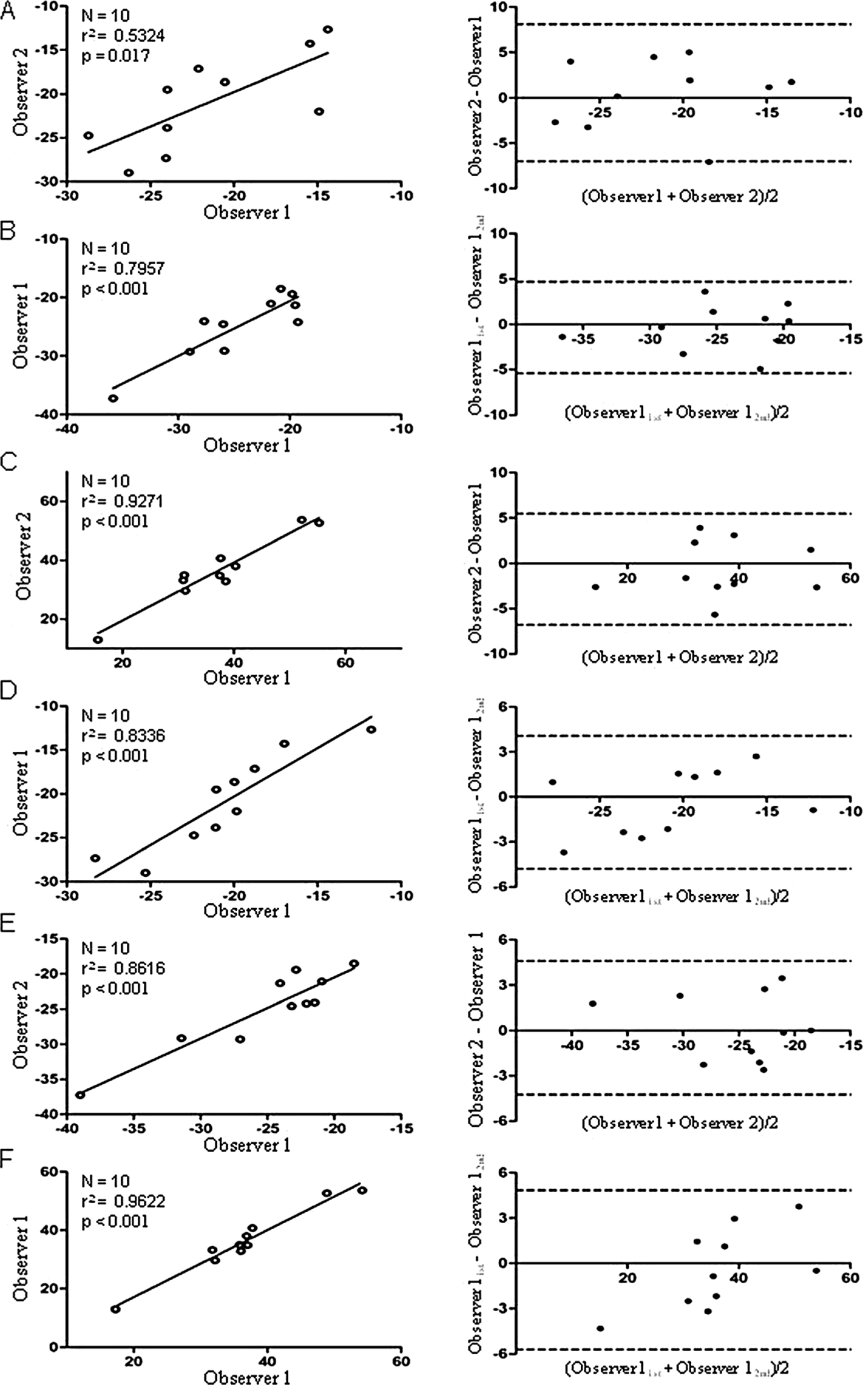
**Scatterplots (left panel) and Bland-Altman plots (right panel).** Comparison between observers (inter- and intra-agreement) at the left ventricular global longitudinal **(A and B)**, circumferential **(C and D)**, and radial strains **(E and F)**. Ninety-five percent limits of agreement are shown as 2 dotted lines in the right panel.

The interobserver and intra-observer agreements concerning left ventricular global longitudinal strain (0.53 and −0.37 for bias, respectively), circumferential strain (−0.35 and 0.17 for bias, respectively), and radial strain (−0.66 and −0.43 for bias, respectively) were satisfactory (Figure 4).

## Discussion

Compared with hypertensive patients without ApHCM, those with hypertension and ApHCM had 1) more left ventricular systolic and diastolic dysfunctions; 2) decreased apical longitudinal and radial strains without a base-to-apex gradient; 3) constantly reduced apical anterior and lateral wall strains irrespective of strain measurement method; 4) independently reduced global longitudinal deformation. Interestingly, these results were obtained from patients with normal left ventricular ejection fractions, highlighting the evidence that alterations of myocardial strain are early contractile abnormalities in hypertensive patients with ApHCM.

The outcomes of patients with ApHCM are not as benign as previously thought [1,2,5,6]. Moon et al. [7] found that the presence of hypertension was the most negative determinant of poor clinical outcomes in patients with ApHCM. Consistent with this report, we also found that ApHCM conferred a negative impact on hypertensive patients in terms of systolic and diastolic functions. A reduced Sm, which indicates impaired myocardial contractility, could represent consequent subclinical systolic dysfunction despite a normal left ventricular ejection fraction. Furthermore, our patients had diastolic dysfunction as evidenced by a dilated left atrium, longer IVRT, and higher diastolic function grade as noted by others [7,19], which suggests that local apical hypertrophy could affect global left ventricular diastolic function. With impaired left ventricular systolic and diastolic functions of ApHCM, the left ventricular stroke volume is consequently reduced despite a normal left ventricular ejection fraction.

In hypertrophic cardiomyopathy patients, longitudinal strain is reduced heterogeneously as assessed by magnetic resonance imaging tagging [20]. Similarly, Yang et al. [12] also found that longitudinal strain is reduced in both septal hypertrophic cardiomyopathy and ApHCM. The subendocardial region contributes primarily to the longitudinal mechanics of the left ventricle. Therefore, the subendocardial layer is vulnerable to the effect of pressure and ischemia that are more commonly observed in hypertrophic cardiomyopathy. Abnormal myocardial capillary density and microvascular dysfunction occur in the hypertrophic myocardial segments of patients with ApHCM [19,21]. These processes cause pronounced relative ischemia in the subendocardial layer, which may result in the formation of fibrotic tissue in the hypertrophic apex of hearts with ApHCM [22]. Normally, left ventricular strains are heterogeneous: both the longitudinal and circumferential strains are higher in the apical and middle segments than basal segments. Continuous shortening in the longitudinal and circumferential direction would result in thickening in the radial direction for mass conservation. Therefore, the apical radial strain is lower, but the longitudinal and circumferential strains are higher than in the basal segment [23,24]. We also found similar findings with further reduced apical radial strain in hypertensive patients with ApHCM than in hypertensive patients without ApHCM. These findings suggest that the hypertrophic apex of hearts with ApHCM is characterized by both morphological and functional abnormalities, which cause the disappearance of the base-to-apex gradient. In addition, we also observed attenuated longitudinal values in the middle ventricular segments relative to the basal segments, in contrast to the base-to-apex gradient in longitudinal values reported by Sun et al. [23]. This finding appears to indicate that the pathologic ventricular hypertrophy extends beyond the apex into the middle and basal ventricular parts of hearts in cases of ApHCM [22,25].

With characteristic insonation angle independence, 2-dimensional speckle tracking echocardiography allows researchers to investigate cardiac mechanics not only globally, but also regionally; as a result, it is uniquely suited for the assessment of left ventricular apical deformation such as that of ApHCM. In the present study, hypertensive patients without ApHCM (control group) had lower than normal strain values, which is similar to the findings of Mizuguchi et al. [26]. Based on the comorbidity of ApHCM in hypertensive patients, the left ventricular mechanics are worse than that in hypertensive patients without ApHCM. Inoue et al. [27] found that left ventricular free walls, as well as hypertrophic septa, are dysfunctional in patients with hypertrophic cardiomyopathy, indicating the importance of the lateral wall function and structure. We also observed constantly reduced apical anterior and lateral wall strains irrespective of strain measurement method, suggesting that the left ventricular apical free walls are the most dysfunctional. The global 2-dimensional longitudinal strain is a surrogate parameter of myocardial fibrosis and cardiac events in patients with hypertrophic cardiomyopathy [28]. Consistent with another study involving patients with decreased functional capacity, [29] we noted that global longitudinal strain was independently associated with hypertension with ApHCM, suggesting that global longitudinal strain is a valuable marker in hypertensive patients with ApHCM. Subendocardial dysfunction is partly responsible for the association because the subendocardial region contributes primarily to the longitudinal strain, and the region is most vulnerable to the effects of pressure and ischemia in ApHCM.

With the electrocardiographic changes, some investigators found that the late and abrupt development of ApHCM occurs at an elderly age [30-32]. The late development of ApHCM in the elderly may indicate a gene-environment interaction required for phenotypic manifestations. The “inducers” making the morphology of ApHCM in the adult patients are not known. Hypertension is associated with ApHCM, and its prevalences in the previous study cohort were 51%–67% [7,32] which is higher than that of the general population [33]. Although it is difficult to prove causality, this raises the possible role of sustained hypertension as an inducer of ApHCM. Therefore, we used hypertension as a baseline comorbidity to study the mechanical effect of ApHCM on left ventricular systolic deformation. Based on the presence of early left ventricular mechanic abnormalities in prehypertension [34], further studies on whether the impact of ApHCM on the left ventricle is localized to or extended beyond the apex would provide more information to understand left ventricular mechanics. Consistent with the prior study on prehypertension and hypertension [34-36], our study showed impaired basal and middle longitudinal strains even in the well-controlled hypertensive patients. With the presence of apical hypertrophy, the global longitudinal and circumferential strains decreased further although the blood pressure control was adequate. These findings indicate that ApHCM might develop progressively and incidentally even in the presence of well-controlled hypertension. Only regular follow-up with electrocardiography and/or echocardiography could identify these patients earlier.

### Limitations

First, the retrospective design limited data analyses in the present investigation. Second, the number of patients included in the present study was small. Further research with a larger number of patients and a prospective design are needed to confirm our findings. Third, this study was conducted at a single tertiary center study. Therefore, our study population might not represent all patients with hypertension and ApHCM. Nonetheless, there are few if any studies using 2-dimensional speckle-tracking echocardiography for evaluating left ventricular mechanics in hypertensive patients with ApHCM. Therefore, our results provide valuable new insights into understanding the effects of pathological apical hypertrophy on left ventricular function in patients with hypertension. Finally, the incomplete coverage of apical hypertrophy might limit the strain analysis in patients with ApHCM.

## Conclusions

ApHCM conferred a negative impact on left ventricular function in hypertensive patients. Reduced apical longitudinal and radial strains without a base-to-apex gradient were present in hypertensive patients with ApHCM. These alterations of myocardial strain are evidence of early contractile abnormalities in hypertensive patients with ApHCM. Among the systolic parameters measured, the global longitudinal strain was an independent predictor of hypertension with ApHCM.

## Consent

Written informed consent was obtained from the patient for the publication of this report and any accompanying images.

## Abbreviations

ApHCM: Apical hypertrophic cardiomyopathy; Am: Mitral annular velocity in late diastole; Em: Mitral annular velocity in early diastole; IVRT: Isovolumic relaxation time; Sm: Mitral annular velocity in peak systole.

## Competing interests

The authors declare that they have no competing interests.

## Authors’ contributions

Y-C Kao analyzed data and drafted the manuscript M-F Lee, C-T Mao, W-S Chen, N-I Yang and W-J Cherng helped design the study and interpret data; M-J Hung designed the study and interpreted the results. All authors read and approved the final manuscript.
